# Construction and case study of a novel lung cancer risk index

**DOI:** 10.1186/s12885-022-10370-4

**Published:** 2022-12-06

**Authors:** Ali Faghani, Lei Guo, Margaret E. Wright, M. Courtney Hughes, Mahdi Vaezi

**Affiliations:** 1 College of Engineering and Engineering Technology, Northern Illinois University, DeKalb, IL USA; 2 School of Interdisciplinary Health Professions, Northern Illinois University, DeKalb, IL USA; 3 University of Illinois Cancer Center, Chicago, IL USA; 4 School of Health Studies, Northern Illinois University, DeKalb, IL USA

**Keywords:** Lung cancer, Risk factors, Analytic hierarchy processes, Meta-analysis, Risk index

## Abstract

**Purpose:**

This study constructs a lung cancer risk index (LCRI) that incorporates many modifiable risk factors using an easily reproducible and adaptable method that relies on publicly available data.

**Methods:**

We used meta-analysis followed by Analytic Hierarchy Process (AHP) to generate a lung cancer risk index (LCRI) that incorporates seven modifiable risk factors (active smoking, indoor air pollution, occupational exposure, alcohol consumption, secondhand smoke exposure, outdoor air pollution, and radon exposure) for lung cancer. Using county-level population data, we then performed a case study in which we tailored the LCRI for use in the state of Illinois (LCRI_IL_).

**Results:**

For both the LCRI and the LCRI_IL_, active smoking had the highest weights (46.1% and 70%, respectively), whereas radon had the lowest weights (3.0% and 5.7%, respectively). The weights for alcohol consumption were 7.8% and 14.7% for the LCRI and the LCRI_IL_, respectively, and were 3.8% and 0.95% for outdoor air pollution. Three variables were only included in the LCRI: indoor air pollution (18.5%), occupational exposure (13.2%), and secondhand smoke exposure (7.6%). The Consistency Ratio (CR) was well below the 0.1 cut point. The LCRI_IL_ was moderate though significantly correlated with age-adjusted lung cancer incidence (*r* = 0.449, *P* < 0.05) and mortality rates (*r* = 0.495, *P* < 0.05).

**Conclusion:**

This study presents an index that incorporates multiple modifiable risk factors for lung cancer into one composite score. Since the LCRI allows data comprising the composite score to vary based on the location of interest, this measurement tool can be used for any geographic location where population-based data for individual risk factors exist. Researchers, policymakers, and public health professionals may utilize this framework to determine areas that are most in need of lung cancer-related interventions and resources.

**Supplementary Information:**

The online version contains supplementary material available at 10.1186/s12885-022-10370-4.

## Introduction

Cancer is the second leading cause of death in the US, with lung cancer accounting for almost one-quarter of these deaths. The American Cancer Society estimates that 236,740 new lung cancers will be diagnosed in 2022, and this disease will claim the lives of more than 130,000 men and women [1]. Numerous studies have examined risk factors for lung cancer, with smoking being the single largest contributor to the disease [2–11]. Other established risk factors include age [12], secondhand smoke exposure [13], environmental exposures (radon [14], indoor and outdoor air pollution [15, 16]), occupational exposures [17], diet [18], alcohol consumption [19], genetic predisposition [20], previous lung disease [21], and arsenic exposure [22]. Many of these risk factors are modifiable, including active smoking and secondhand smoke exposure, environmental exposures, occupational exposures, alcohol consumption, and diet [23].

Although many studies have investigated associations between individual risk factors and lung cancer risk or mortality [20–32], less is known about how these factors interact to influence the development and progression of the disease. Some studies have examined interactions between smoking and one other risk factor, such as radon, alcohol consumption, family history, previous lung disease, or some component of diet [33]. To our knowledge, there are few, if any, studies that simultaneously investigated the contribution of more than two modifiable risk factors for lung cancer. This may be because epidemiologic studies are often limited in their ability to consider multiple factors simultaneously, given limited sample sizes and ranges of exposures within their study populations [34].

To address this gap, we constructed a Lung Cancer Risk Index (LCRI) that incorporates several modifiable risk factors using Meta-Analytic Hierarchy Process (Meta-AHP). While this approach has been used in the soil science field [35], it has not been commonly employed in the health sciences. Meta-AHP may be superior to a traditional principal component analysis approach because Meta-AHP can effectively extract essential variables and assign weights more precisely. We tailored this index for use in a case study of the state of Illinois; the LCRI_IL_ was created using publicly available county-level data for all 102 Illinois counties. We then evaluated the correlation between the LCRI_IL_ and reported lung cancer incidence and mortality rates. We provide researchers with an easily reproducible and adaptable method that uses publicly available data to generate a composite measure that integrates multiple modifiable risk factors for lung cancer. This measure can be tailored for any geographic area and is potentially widely applicable. Public health officials and policymakers may consider using this measure when making decisions regarding lung cancer-related interventions and resource allocation in their communities.

## Methods

**Fig. 1:**
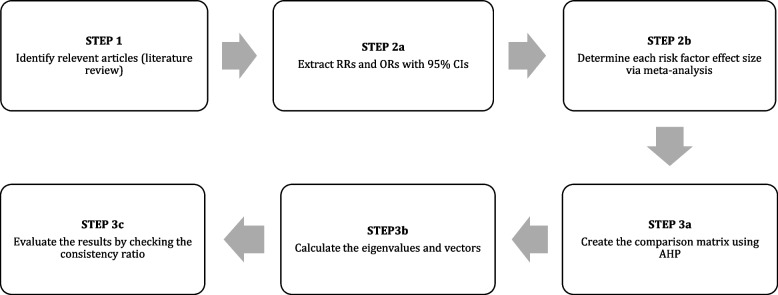
Flowchart showing the process used to generate the Lung Cancer Risk Index (LCRI). AHP = Analytic Hierarchy Process, CI = confidence interval, OR = odds ratio, RR = relative risk

Figure 1 shows the process that we used to generate the lung cancer risk index (LCRI). Each step in the figure is explained in detail below.

### Step 1: identify relevant articles: search strategy and article selection

Using the Preferred Reporting Items for Systematic Reviews and Meta-Analyses (PRISMA) guidelines [36], we conducted searches of PubMed (including MEDLINE) and Google Scholar for full-length articles that were published between January 1990 and April 2021. We utilized the following keyword strings to capture relevant studies: “lung cancer” in conjunction with one of the following—“smoking,” “passive smoking,” “secondhand smoke,” “radon,” “occupation,” “air pollution,” “alcohol consumption,” or “risk factors.” We did not include diet in our index because the World Cancer Research Fund (WCRF) and American Institute for Cancer Research (AICR) consider there to be “limited evidence” that diet is a risk factor for lung cancer [37]. We chose to exclude arsenic exposure from our index because the US public water supply levels are kept below 50 µg/L [38, 39], which is far below concentrations associated with increased lung cancer risk [22, 40]. Nevertheless, researchers in other countries should consider adding arsenic to an LCRI adapted for use in their locations. We assessed the quality of the articles included in the present study using appraisal checklists and criteria of quality recommended by JBI (formerly known as "Joanna Briggs Institute"), an international organization focused on improving evidence as it relates to the feasibility, appropriateness, meaningfulness, and effectiveness of healthcare interventions [41].

**Fig. 2:**
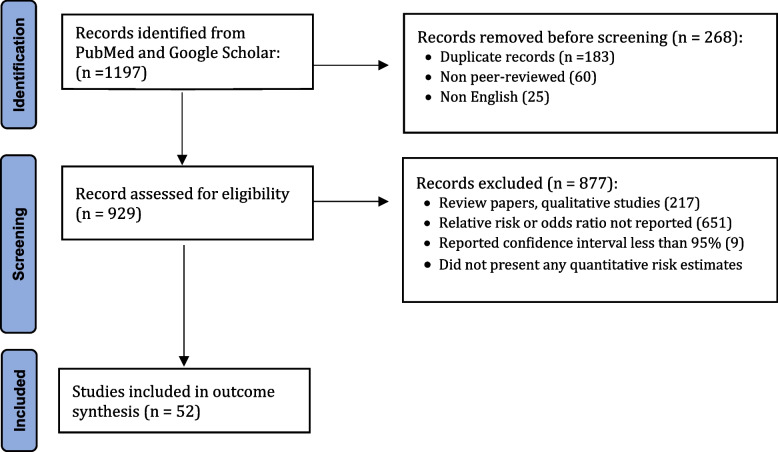
Flowchart of search methodology and article selection

As shown in Fig. 2, the initial literature search yielded 1197 articles. We removed 268 articles that were duplicates, not peer-reviewed prior to publication, or written in languages other than English. We then reviewed the abstracts of the 929 remaining articles and applied the study inclusion criteria: (1) randomized controlled trial, prospective cohort study, retrospective cohort study, case-cohort study, case–control study, or nested case–control study; (2) reported the relative risk (RR) or odds ratio (OR) associated with *increased* risk (i.e., RR or OR > 1, which is a requirement of the Analytic Hierarchy Process (AHP) model); and (3) reported 95% confidence intervals (CIs). After excluding 877 articles that did not meet the inclusion criteria specified above, at least two researchers reviewed the full text of the remaining 52 manuscripts [42].

### Steps 2a / 2b: meta-analysis

The second step in creating our index was to extract the adjusted OR and RR from all 52 articles for each lung cancer risk factor examined (Additional file Table 1). Next, a weighted average of study-specific estimates using inverse variance weights was derived for each risk factor [43] to increase the accuracy of outcomes [44, 45]. The potential for publication bias was evaluated by funnel plots and the methods described by Egger et al. [46] and Begg et al. [47]. Using a random-effects model, we analyzed the studies and considered heterogeneity and within-study variance [48]. We evaluated heterogeneity using Cochrane’s Q-statistic [49] and the *I*^2^ inconsistency statistical tests [50].

We considered the OR to be a good approximation of the RR for our analysis, which is reasonable when the outcome is rare [51]. We used the OR and the logOR and calculated standard errors (SEs) as data points for the meta-analysis. All statistical manipulations were conducted using the meta-analysis package for R (metaphor Version 2, MA, USA).

### Steps 3a-3c: Analytic Hierarchy Process (AHP)

The third step in creating our index was to use the results of our meta-analysis as inputs for the AHP analysis and to generate weights for each risk factor. AHP is one of the most widely used Multi-Criterion Decision Making (MCDM) methods [52] and has been increasingly implemented in health care, including cancer research [53–57]. AHP can quantitatively prioritize risk factors by producing weights for each factor, making it an ideal method to apply in this study. For each included modifiable risk factor, we used the OR derived from our meta-analysis as input variables in the AHP. Using the values from meta-analysis and the assessment matrix, we created the pair-wise comparison matrix (i.e., a matrix to compare risk factors in pairs to evaluate their relative importance). We created an assessment matrix with numbers that pair with different importance levels. For example, 1, 3, 5, 7, and 9 pair with equal, weak, obvious, intense, and extreme importance, while 2, 4, 6, and 8 pair with intermediate importance, respectively [58] (Additional file Table 1).

The relative importance of smoking versus all other included risk factors was assigned considering the assessment matrix. This step was then repeated for all other remaining risk factors. Next, an *n* by *n* matrix was created where *n* represented the number of modifiable risk factors. Next, we solved the linear system, where A is the coefficient matrix using Eq. 1:

1 $$AX=\lambda X or \left(A-\lambda {I}_{n}\right)X=0$$

where *A* is the comparison matrix of order n, and *λ* is one of its eigenvalues. *X* represents the eigenvector of *A* associated with *λ*, and *A-λI*_*n*_ represents the matrix coefficient. We used MATLAB (MathWorks, Massachusetts, USA) to calculate the eigenvalues and eigenvectors of the matrix [59]. Then we used the derived eigenvector to specify the weights of each risk factor where the eigenvector represented the index coefficient. Next, we estimated the contribution of each risk factor to lung cancer. We then calculated the z score and considered the z score as the corresponding value in the index. Finally, z-scores were converted to percentiles for mapping purposes.

We used the Consistency Ratio (CR) to verify the reliability of our results. To do this, we first calculated the Consistency Index (*CI*_*1*_) using the following equation:

2 $${CI}_{1}= (\lambda max-n)/(n-1)$$

where $\lambda max$ was the maximum eigenvalue and *n* represented the order of the matrix. Accordingly, the CR was calculated by dividing the *CI*_*1*_ by the index for the corresponding Random Index (RI) using the following equation:

3 $${CI}_{1}= {CI}_{1}/RI$$

Saaty [60] has presented the values for *RI* considering the matrix size. Also, Saaty [60] suggested that the *CR* needs to be less than 0.1 to produce consistent results.

## Results

As shown in Table 1, the process that we used to create the LCRI yielded the highest weight for active smoking (46.1%) and the lowest weight for radon exposure (3.0%). The CR of the AHP analysis for the present study was 0.07, well below the 0.1 cut point that demonstrates consistency of the analysis.

**Table 1:** Overall effect size and final weights for modifiable risk factors included in the Lung Cancer Risk Index (LCRI)

No	Risk factor	Overall effect size from meta-analysis	Weights used in LCRI (%)
**1**	**Active smoking**	8.63	46.1
**2**	**Indoor air pollution**	1.76	18.5
**3**	**Occupational exposure**	1.60	13.2
**4**	**Alcohol consumption**	1.45	7.8
**5**	**Secondhand smoke exposure**	1.43	7.6
**6**	**Outdoor air pollution**	1.25	3.8
**7**	**Radon exposure**	1.24	3.0
	**Total**		100

We used the weights in Table 1 to produce the LCRI:

4 $$LCRI=0.461{A}_{1}+0.185{A}_{2}+0.132{A}_{3}+0.078{A}_{4}+0.076{A}_{5}+0.038{A}_{6}+0.030{A}_{7}$$

where A_1_ to A_7_ represent each included modifiable risk factor, as listed in Table 1. It should be noted that *A*_*1*_ to *A*_*7*_ can be values of 0 or 1, where 0 indicates the corresponding risk factor was not in effect and 1 indicates the corresponding risk factor was in effect (i.e., 0 = no exposure and 1 = exposure / risk exists). We calculated the corresponding z score for each geographical area (e.g., if the emitted air pollution for a county is X tons/year, the corresponding value for *A*_*6*_ would be the corresponding z score which is dependent on the average and variance of emitted air pollution for that specific county compared to all other counties in any state). Developed countries such as the US do not rely on major sources of household air pollution—kerosene, wood, or coal—to generate heat [61, 62], so A_2_ is assigned a value of 0 for individuals living in these countries. The $\mathrm{LCRI}$ can take any value between 0 and 1: an LCRI value of 0 means no predicted lung cancer risk (A_1_ to A_7_ all equal 0), and an LCRI value of 1 represents the highest possible predicted risk of lung cancer.

## Case study

We test the adaptability and utility of the LCRI in a case study performed using data for our home state of Illinois. In this case study, we constructed the LCRI_IL_ – a version of the LCRI that reflects the available population-level data in our state. IL is comprised of 102 counties, some of which are urban and many of which are rural. Forty percent of the state’s population resides in Cook County – home to the City of Chicago. Cook County is the second-most populous county in the nation, with more than 5.2 million racially and ethnically diverse residents [63].

Our first step in creating the LCRI_IL_ was to collect all necessary risk factor data from publicly available data sources. For all counties, we extracted data for 2014–2019 for active smoking (percentage of adults who are current smokers), radon exposure (pCi/L), outdoor air pollution (concentration of fine particulate matter (PM2.5)), and alcohol consumption (percentage of adults reporting binge or heavy drinking in past 30 days) [64, 65]. There were no publicly available county-level data for secondhand smoke exposure or occupational exposures, so those risk factors were dropped from the LCRI_IL_.

The second step in creating the LCRI_IL_ was to generate weights for each available risk factor using the previously described methods (see Methods, Steps 3a-3c). The weights used in the LCRI_IL_ were 0.70 for active smoking, 0.14 for alcohol consumption, 0.095 for outdoor air pollution, and 0.057 for radon exposure. The corresponding equation to derive the LCRI_IL_ is:

5 $${LCRI}_{IL}=0.701{B}_{1}+0.147{B}_{2}+0.095{B}_{3}+0.057{B}_{4}$$

where *B*_*1*_ to *B*_*4*_ represent active smoking, alcohol consumption, outdoor air pollution, and radon exposure, respectively. The CR of the AHP analysis for the case study was 0.04, which indicated the consistency of the analysis.

**Fig. 3:**
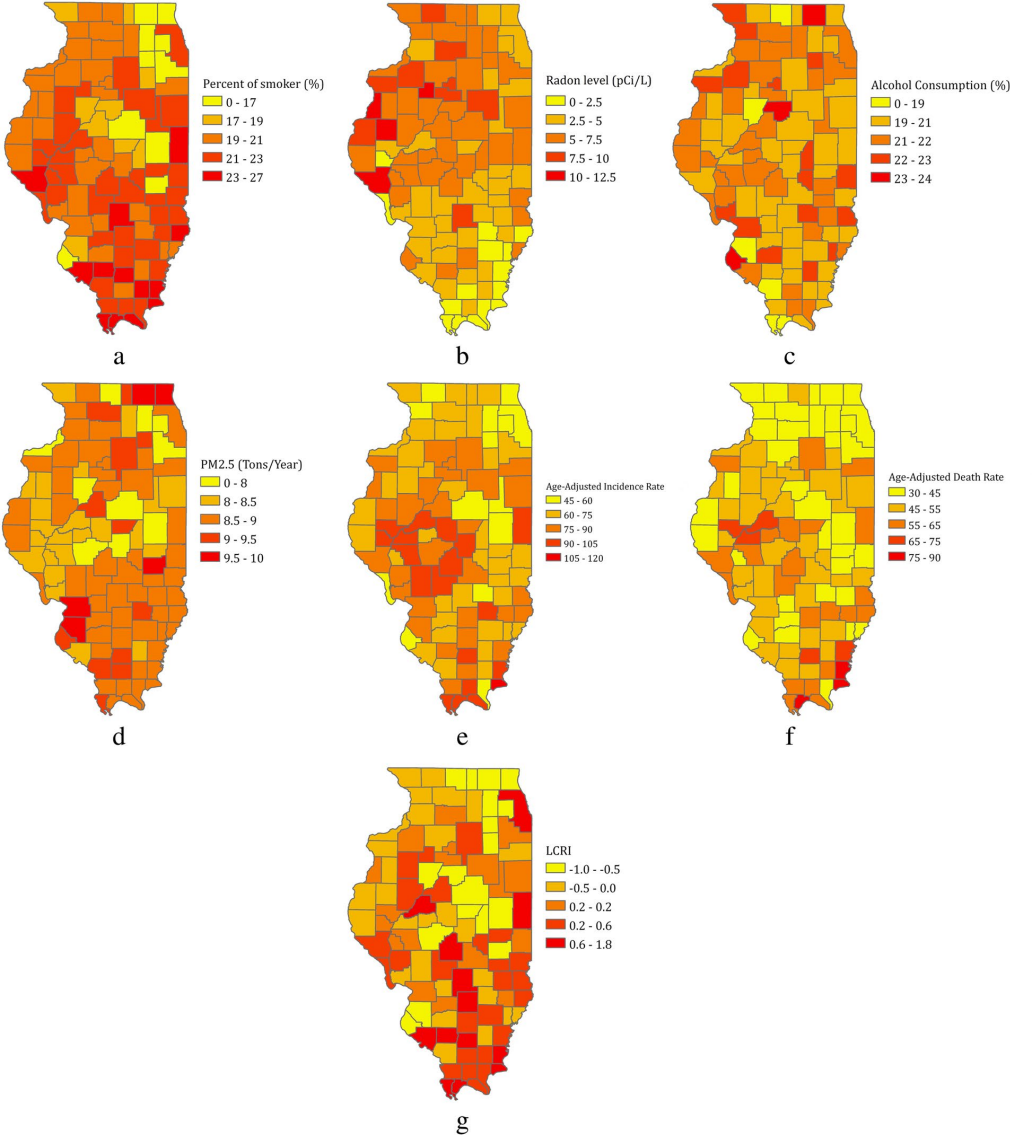
Maps showing the prevalence of risk factors for each of Illinois’ 102 counties: **a**) active smoking (adults, 2014–2019), **b**) radon exposure (2014–2019), **c**) excess alcohol consumption (adults, 2014–2019), **d**) outdoor air pollution (PM2.5, 2014–2019), **e**) Age-adjusted lung cancer incidence rates (2014–2018), **f**) Age-adjusted lung cancer mortality rate (2014–2018), **g**) LCRI percentile

Figure 3 shows the prevalence of each individual risk factor that was included in the LCRI_IL_, as well as lung cancer outcomes [66], by county across Illinois. There is substantial heterogeneity for each risk factor across the state. Among the top 28 counties that have the highest lung cancer incidence and / or mortality rates, eight are also among the top 20 LCRI_IL_ counties. These eight counties are predominantly located in rural areas (as defined by the US census, [63]) of Southern and Southeastern Illinois, though one is an urban county located on the east side of the state. Notably, Cook County had the highest LCRI_IL_ score but among the lowest lung cancer incidence and mortality rates.

Table 2 presents Pearson correlation coefficients between the LCRI_IL_ z scores, active smoking, and lung cancer incidence and mortality rates. The correlation coefficients between the LCRI_IL_ and lung cancer incidence and mortality were 0.45 and 0.50, respectively, with both *p*-values < 0.05. The correlation coefficient between the LCRI_IL_ and active smoking was high at 0.87, which was expected given that this risk factor had the highest assigned weight in the index.

**Table 2:** Results of Pearson correlation test between LCRI_IL_, percentage of active smokers, age-adjusted lung cancer incidence rate, and age-adjusted lung cancer mortality rate

	**LCRI**_**IL**_** z score**	**Active Smoker (%)**	**Age-Adjusted lung cancer Incidence Rate (2014–2018)**	**Age-Adjusted lung cancer mortality Rate (2014–2018)**
LCRI_IL_ z score	1	.869^a^	.449^a^	.495^a^
Active Smoker (%)		1	.498^a^	.418^a^
Age-Adjusted lung cancer Incidence Rate (2014–2018)			1	.812^a^
Age-Adjusted lung cancer mortality Rate (2014–2018)				1

^a^Correlation is significant at the 0.05 level (2-tailed)

In sensitivity analyses, we examined the magnitude of the correlation coefficient for each component of the LCRI_IL_ in relation to lung cancer incidence and mortality rates. The correlation coefficient was only statistically significant for active smoking, and the magnitude and significance were similar to that of the LCRI_IL_ (Table 3). In an additional sensitivity analysis, alcohol consumption was dropped from the LCIR_IL_ – since it is so highly correlated with active smoking – and the resulting index showed similar correlation with lung cancer incidence and mortality rates (0.496 and 0.545, respectively) as compared to the original index.

**Table 3:** Sensitivity analysis of individual components of the LCRI_IL_ in relation to lung cancer outcomes in Illinois

		Age-adjusted IL lung cancer incidence rate (2014—2018)	Age-adjusted lung cancer mortality rate (2014—2018)
Active smoking	Pearson Correlation	.498^a^	.418^a^
	Sig. (2-tailed)	0.000	0.000
	N	102	102
Air pollution (PM2.5)	Pearson Correlation	0.066	0.055
	Sig. (2-tailed)	0.510	0.581
	N	102	102
Radon (pCi/L)	Pearson Correlation	-.124	-.126
	Sig. (2-tailed)	0.510	0.581
	N	102	102
Excess alcohol consumption (%)	Pearson Correlation	-.188	-0.063
	Sig. (2-tailed)	0.825	-.187
	N	102	102

^**a**^Correlation is significant at the 0.05 level (2-tailed)

## Discussion

We created a novel lung cancer risk index (LCRI) that integrates multiple modifiable risk factors into one measure. Active smoking is the predominant risk factor for lung cancer and is linked with 80–90% of lung cancer deaths [25]. As expected, smoking received the highest weight in both our original index (LCRI: 46.1%) and the one that we adapted for use in the state of Illinois (LCRI_IL_: 70.1%). Conversely, radon exposure had the lowest weight in each index (LCRI: 3%, LCRI_IL_: 5.7%).

Previous studies have largely focused on associations between individual risk factors and lung cancer outcomes [11, 13, 25, 29]. However, there are laboratory, animal, and human data showing that risk factors interact with each other to affect cancer outcomes [67–69]. For example, Wu et al. [67] reviewed and highlighted the evidence that cancer causation is multifactorial and suggested that researchers consider the contributions of individual factors and their joint effects on cancer burden. Li et al. showed that gene-smoking interactions play important roles in the etiology of lung cancer 68]. Our index represents an attempt to address these known interactions by using population-based data to capture the combined impact of multiple risk factors for lung cancer into one measure.

Hot spots identified by our index share similar distribution patterns of risk factors from the geospatial analysis. Interestingly, Cook County has the highest LCRI_IL_ despite low adjusted lung cancer incidence and mortality rates. Although Cook County has moderate to high levels of alcohol consumption, Fine Particulate Matter 2.5, and air pollution, it also has a high ratio of primary care physicians to the population (1050:1, ranked 8th in IL), suggesting greater availability of healthcare resources. This may explain the discordance between Cook County’s LCRI_IL_ and lung cancer incidence and mortality rates. Counties with high LCRI_IL_ and high lung cancer incidence or mortality rates are mostly in the rural area of the state with fewer available healthcare resources [70]. This echoes findings from recent studies that cancer mortality rates associated with modifiable risks were higher in rural compared with urban populations [71, 72].

Cancer is a heterogeneous disease [73] with many risk factors at individual and social levels. Our model included the factors studied in the literature where the studies met the criteria for inclusion (e.g., being a modifiable risk factor, having an OR or RR, etc.); however, it is important to note that other non-modifiable factors such as age, gender, and race have been shown to also be strongly associated with lung cancer’s incidence and mortality rates [74]. Nevertheless, the study offers a useful framework that health policymakers and researchers can use to identify and examine potential lung cancer risk factors for their geographical areas.

Our study has several strengths. First, to our knowledge, ours is the first study to use meta-analysis in combination with AHP to create a composite risk index for a specific cancer. Second, our model summarized complex and multi-dimensional factors to provide a tool for use by healthcare decision-makers. Our index includes several major and minor modifiable risk factors rather than a single biomedical factor. Third, our study presents a new approach where researchers and policymakers can utilize databases (e.g., U.S. Centers for Disease Control & Prevention’s Behavioral Risk Factor Surveillance System, U.S. Environmental Protection Agency’s Office of Air Quality Planning and Standards, etc.) at multiple geographic levels to identify areas that may benefit from resource allocation and public health interventions. Additionally, a Meta-AHP approach could potentially be combined with machine learning and deep learning models [75, 76] to analyze risk factors and predict health outcomes more accurately.

There were several limitations to this study. First, the AHP approach only allows for the inclusion of risk estimates greater than 1. As a result, we could not include protective behaviors such as fruit and vegetable consumption in our index. Second, AHP relies directly and exclusively on the magnitude of a single risk estimate generated from the meta-analysis, which is likely an underestimate because the model does not allow for variation in exposure prevalence by region. As an example, radon is widely considered to be the second leading cause of lung cancer, behind cigarette smoking [77]. However, as shown in Table 1, this risk factor received the lowest weight in the index because the risk estimate from the meta-analysis was only 1.24–the smallest magnitude of any factor examined. Third, we could not include secondhand smoke and occupational exposures in our tailored LCRI_IL_ index because county-level data in Illinois are not publicly available for these two factors. We also did not include non-modifiable risk factors such as age, gender, and race. Fourth, because alcohol consumption and tobacco smoking are strongly correlated, the confounding effect of smoking may impact the weight of alcohol consumption in the LCRI. However, when we removed alcohol consumption from LCRI_IL_ in a sensitivity test, the resulting index showed similar correlation to lung cancer outcomes. Future research is needed to examine the effect the strong correlation between smoking and alcohol has on the LCRI. Fifth, we imposed a single cut point for each risk factor in our models, while, in actuality, some risk factors may exhibit curvilinear or other types of relationships with cancer outcomes. Finally, the meta-analysis was limited to literature published in 1990 and beyond, and therefore did not capture earlier studies.

## Conclusion

We generated a lung cancer risk index that incorporated several modifiable risk factors into one composite score. The index was driven heavily by active smoking, as expected. In addition, the index was modestly correlated with lung cancer outcomes in a case study conducted in Illinois, demonstrating its adaptability and potential utility in numerous geographic locations and potentially in many different fields. Future refinements to the index could include adding other modifiable risk factors, examining the impact of non-modifiable risk factors such as age, gender, and race / ethnicity in the LCRI, performing geographical cluster analysis, and incorporating other health behavior factors in AHP-based cancer risk factor models for lung cancer or other health outcomes.

## Supplementary Information

**Additional file 1.**

## Data Availability

All data generated or analyzed during this study are included in this published article and its supplementary information files.
