# Supplementary material for: Construction and case study of a novel lung cancer risk index
Source: BMC Cancer. 2022 Dec 6;22:1275. doi: 10.1186/s12885-022-10370-4 (PMC9724373; doi:10.1186/s12885-022-10370-4)
Supplement: Supplementary file 1 — Additional file 1. [file 12885_2022_10370_MOESM1_ESM.docx]

**Appendix**

**Table of Content**

| **Table 1** Point estimates for selected modifiable lung cancer risk factors **………………………………………** | 2 |
| --- | --- |
| **Table 2** LCRI_IL_ Z Score, Percentile, and Rank for Each County In Illinois**…………………………………….** | 5 |
| **More Details regarding AHP Procedure ……………………………………………………………………..** | 5 |
| **Table 3** The scale and deﬁnition of the assessment matrix .**………………………………………………….** | 7 |
| **Table 4:** The pairwise comparison matrix **……………………………………………………………………...** | 7 |
| **References ……………………………………………………………………………………………………** | 7 |

**Table 1** Point estimates for selected modifiable lung cancer risk factors

| Risk factor | Gender | Type/Content | Measure | Point estimate | 95% CI Lower limit | 95% CI Upper limit | Ref |  |
| --- | --- | --- | --- | --- | --- | --- | --- | --- |
| Smoking | Male |  | OR | 7.82 | 4.59 | 13.30 | [1] |  |
| Smoking | Female |  | OR | 11.76 | 7.50 | 18.42 | [1] |  |
| Smoking (United States smokers) | Both |  | OR | 40.4 | 21.8 | 79.6 | [2] |  |
| Smoking (Japanese smokers) | Both |  | OR | 3.5 | 1.60 | 7.50 | [2] |  |
| Smoking | Male |  | OR | 9.6 | 5.64 | 16.30 | [3] |  |
| Smoking | Female |  | OR | 27.9 | 14.9 | 52.0 | [3] |  |
| Smoking | Female |  | RR | 3.4 | 1.75 | 6.61 | [4] |  |
| Smoking | Male |  | RR | 4.39 | 3.92 | 4.92 | [5] |  |
| Smoking | Female |  | RR | 2.79 | 2.44 | 3.20 | [5] |  |
| Smoking | Both |  | OR | 14.9 | 12.3 | 18.1 | [6] |  |
| Smoking | Male |  | OR | 5.0 | 2.0 | 12.7 | [7] |  |
| Smoking | Both |  | OR | 9.40 | 6.9 | 12.8 | [8] |  |
| Smoking | Female |  | OR | 13.6 | 12.3 | 15.1 | [9] |  |
| Smoking | Male |  | OR | 11.3 | 10.2 | 12.4 | [9] |  |
| Smoking | Female |  | OR | 8.94 | 7.54 | 10.6 | [10] |  |
| Smoking | Female |  | OR | 4.87 | 1.34 | 17.75 | [11] |  |
| Radon exposure | Both | (200+ Bq/m^3^) | OR | 1.29 | 0.98 | 1.70 | [12] |  |
| Radon exposure | Both | (150-199 Bq/m^3^) | OR | 1.19 | 0.86 | 1.66 | [12] |  |
| Radon exposure | Both | (100-149 Bq/m^3^) | OR | 1.22 | 0.88 | 1.69 | [12] |  |
| Radon exposure – North America | Both | ( up to 100 Bq/ m^3^) | OR | 1.106 | 1 | 1.28 | [13] |  |
| Radon exposure – China | Both | (up to 100 Bq/m^3^) | OR | 1.139 | 1.01 | 1.37 | [13] |  |
| Radon exposure | Both | (100 Bq/m^3^) | OR | 1.33 | 1.01 | 1.36 | [14] |  |
| Radon exposure | Both |  | OR | 1.73 | 1.27 | 2.35 | [15] |  |
| Radon exposure – Germany | Both | (up to 80 Bq/m^3^) | OR | 1.59 | 1.08 | 2.27 | [16] |  |
| Radon exposure – Germany | Both | (above 80 Bq/m^3^) | OR | 1.93 | 0.99 | 3.77 | [16] |  |
| Radon exposure | Both | 50-80 Bq/m^3^ | RR | 1.08 | 0.79 | 1.47 | [17] |  |
| Radon exposure | Both | 80-140 Bq/m^3^ | RR | 1.18 | 0.86 | 1.61 | [17] |  |
| Radon exposure | Both | above 140 Bq/m^3^ | RR | 1.44 | 1 | 2.06 | [17] |  |
| Occupational exposure | Both | (welding fumes) | OR | 2.50 | 1.0 | 6.5 | [8] |  |
| Occupational exposure | Both | (asbestos) | OR | 1.50 | 0.5 | 4.4 | [8] |  |
| Occupational exposure | Both | (wood dust) | OR | 1.90 | 1.2 | 3.1 | [8] |  |
| Occupational exposure | Both | (diesel exhaust) | OR | 3.10 | 2.1 | 4.5 | [8] |  |
| Occupational exposure | Both |  | OR | 1.60 | 1.4 | 2.1 | [18] |  |
| Occupational exposures | Both |  | OR | 2.10 | 1.3 | 3.3 | [18] |  |
| Occupational exposure | Both | (crystalline silica) | OR | 1.37 | 1.14 | 1.65 | [19] |  |
| Occupational exposure | Both | (crystalline silica) | OR | 1.41 | 1.22 | 1.62 | [20] |  |
| Occupational exposure | Both | (diesel exhaust) | OR | 1.43 | 1.23 | 1.67 | [20] |  |
| Occupational exposure | Both | (polycyclic aromatic hydrocarbons) | OR | 1.53 | 1.14 | 2.04 | [20] |  |
| Occupational exposure | Both | (asbestos) | OR | 1.78 | 0.94 | 3.36 | [21] |  |
| Occupational exposure (Low concentrations) | Both | (asbestos) | OR | 1.17 | 0.92 | 1.5 | [22] |  |
| Occupational exposure (medium or high concentrations) | Both | (asbestos) | OR | 2.16 | 1.21 | 3.88 | [22] |  |
| Second hand smoking (highly exposed) | Both |  | RR | 2.01 | 1.33 | 2.60 | [23] |  |
| Second hand smoking | Both |  | OR | 1.26 | 1.06 | 1.47 | [24] |  |
| Second hand smoking | Female |  | OR | 1. 31 | 0.99 | 1.72 | [25] |  |
| Second hand smoking | Both |  | RR | 1.08 | 0.6 | 1.94 | [26] |  |
| Second hand smoking | Both |  | RR | 1.05 | 0.6 | 1.86 | [27] |  |
| Second hand smoking | Female |  | OR | 2.95 | 1.32 | 6.57 | [11] |  |
| Second hand smoking | Both |  | OR | 1.57 | 1.07 | 2.31 | [28] |  |
| Second hand smoking | Both |  | RR | 1.9 | 1.0 | 3.50 | [29] |  |
| Second hand smoking | Female |  | RR | 1.3 | 1.0 | 1.7 | [30] |  |
| Second hand smoking | Female |  | OR | 1.3 | 0.7 | 1.5 | [31] |  |
| Outdoor air pollution | Both | (pesticides) | OR | 5.10 | 3.1 | 8.3 | [8] |  |
| Outdoor air pollution | Both |  | RR | 1.25 | 1.18 | 1.32 | [32] |  |
| Outdoor air pollution | Both | (mortality) | RR | 1.23 | 1.16 | 1.30 | [32] |  |
| Outdoor air pollution | Both | (diesel) | OR | 3.10 | 2.1 | 4.5 | [8] |  |
| Outdoor air pollution | Both |  | OR | 1.46 | 0.89 | 2.40 | [33] |  |
| Outdoor air pollution (low concentrations) | Both | (nitrogen dioxide) | OR | 1.14 | 0.78 | 1.67 | [33] |  |
| Outdoor air pollution (high concentrations) | Both | (nitrogen dioxide) | OR | 1.30 | 1.02 | 1.66 | [33] |  |
| Outdoor air pollution | Both | (PM2.5) | OR | 1.29 | 0.95 | 1.76 | [34] |  |
| Outdoor air pollution | Both | (nitrogen dioxide) | OR | 1.34 | 1.07 | 1.69 | [34] |  |
| Outdoor air pollution | Both | (nitrogen dioxide) | OR | 1.3 | 1.02 | 1.66 | [35] |  |
| Outdoor air pollution | Both | (PM10) | OR | 1.05 | 0.65 | 1.69 | [35] |  |
| Outdoor air pollution | Both | (sulfur dioxide) | OR | 1.15 | 0.92 | 1.43 | [35] |  |
| Outdoor air pollution | Both | (PM2.5) | RR | 1.14 | 1.04 | 1.23 | [36] |  |
| Outdoor air pollution | Both | (Nox) | RR | 1.08 | 1.02 | 1.15 | [37] |  |
| Outdoor air pollution | Both | (Sox) | RR | 1.01 | 0.94 | 1.08 | [37] |  |
| Outdoor air pollution | Both | (PM10) | RR | 1.66 | 1.21 | 2.27 | [38] |  |
| Outdoor air pollution | Both | (Nox) | RR | 1.10 | 0.97 | 1.23 | [39] |  |
| Outdoor air pollution | Both | (Sox) | RR | 1.01 | 0.98 | 1.03 | [39] |  |
| Indoor air pollution | Both | HAP exposure | OR | 1.77 | 1 | 3.14 | [40] |  |
| Indoor air pollution | Both | (PAH25) | OR | 2.21 | 1.67 | 2.87 | [41] |  |
| Indoor air pollution | Both | (NO2) | OR | 2.06 | 1.19 | 3.49 | [41] |  |
| Indoor air pollution (ex-smokers) | Both |  | OR | 4.30 | 2.7 | 6.8 | [42] |  |
| Indoor air pollution | Both | (cooking only) | OR | 1.15 | 0.97 | 1.37 | [43] |  |
| Indoor air pollution | Both | (heating and cooking) | OR | 1.17 | 1.01 | 1.37 | [43] |  |
| Indoor air pollution | Female | wood or straw as cooking fuels | OR | 1.77 | 1.08 | 2.91 | [28] |  |
| Indoor air pollution | Both | Coal consumption (heating and cooking) | OR | 1.29 | 1.03 | 1.61 | [44] |  |
| Indoor air pollution | Female | cooking oil | OR | 2.54 | 1.40 | 4.30 | [45] |  |
| Indoor air pollution | Female | Coal consumption (heating and cooking) | OR | 1.3 | 0.3 | 5.80 | [31] |  |
| Alcohol consumption | Both | (>60 g/day) | OR | 1.44 | 1.01 | 2.07 | [46] |  |
| Alcohol consumption | Both | (>20 g/day) | OR | 1.42 | 1.06 | 1.90 | [47] |  |
| Alcohol consumption | Both |  | OR | 1.60 | 1.00 | 2.04 | [48] |  |
| Alcohol consumption | Both | (white wine) | OR | 1.20 | 1.01 | 1.42 | [49] |  |
| Alcohol consumption | Both | (alcoholism) | RR | 2.40 | 2.29 | 2.51 | [50] |  |
| Alcohol consumption | Both | Above 9 drinks/month | RR | 1.1 | 0.7 | 1.6 | [48] |  |
| Alcohol consumption | Both | Above 1 drink/day | RR | 1.9 | 1.0 | 3.4 | [51] |  |
| Alcohol consumption | Both | Above 0.5 drink/week | RR | 1.1 | 0.6 | 2.1 | [52] |  |
| Alcohol consumption | Both | Above 7 drinks/week | RR | 1.2 | 0.8 | 1.7 | [53] |  |

OR = Odds Ratio

RR = Relative Risk

**Table 2** LCRI_IL_ Z Score, Percentile, and Rank for each county in illinois

| County Name | LCRI_IL_ Z-Score | LCRI_IL_ Percentile | LCRI_IL_ Rank | County Name | LCRI_IL_  Z-Score | LCRI_IL_ Percentile | LCRI_IL_ Rank |
| --- | --- | --- | --- | --- | --- | --- | --- |
| Cook | 1.7962 | 0.99 | 1 | Iroquois | 0.0459 | 0.55 | 52 |
| Mason*, ** | 1.7875 | 0.98 | 2 | Brown* | 0.0392 | 0.55 | 53 |
| Marion | 1.373 | 0.89 | 3 | Morgan* | 0.0349 | 0.53 | 54 |
| Alexander*, ** | 1.2003 | 0.89 | 4 | Putnam | 0.0236 | 0.51 | 55 |
| Fayette | 1.0496 | 0.89 | 5 | Will | 0.0025 | 0.5 | 56 |
| Randolf | 1.0128 | 0.88 | 6 | Ogle | -0.028 | 0.5 | 57 |
| Hardin*, ** | 0.8886 | 0.87 | 7 | Mercer | -0.03 | 0.49 | 58 |
| Gallatin*, ** | 0.8716 | 0.82 | 8 | Cass*, ** | -0.033 | 0.48 | 59 |
| Perry | 0.7656 | 0.78 | 9 | Hamilton | -0.051 | 0.47 | 60 |
| Frankin*, ** | 0.7387 | 0.78 | 10 | Hancock | -0.052 | 0.46 | 61 |
| Vermilion*, ** | 0.7199 | 0.77 | 11 | Macoupin* | -0.057 | 0.46 | 62 |
| Pulaski*, ** | 0.6826 | 0.77 | 12 | Wabash | -0.074 | 0.45 | 63 |
| Christian* | 0.6754 | 0.76 | 13 | Edwards | -0.097 | 0.45 | 64 |
| Pike | 0.5924 | 0.76 | 14 | Washington | -0.099 | 0.44 | 65 |
| Greene | 0.578 | 0.75 | 15 | Carroll | -0.106 | 0.44 | 66 |
| Stark | 0.5628 | 0.75 | 16 | Stephenson | -0.121 | 0.44 | 67 |
| Douglass | 0.5546 | 0.74 | 17 | Whiteside | -0.167 | 0.43 | 68 |
| Lawrence** | 0.5511 | 0.73 | 18 | Bond | -0.207 | 0.43 | 69 |
| Saline** | 0.5459 | 0.73 | 19 | Effingham | -0.231 | 0.43 | 70 |
| Jasper | 0.5278 | 0.71 | 20 | Adams | -0.233 | 0.42 | 71 |
| Crawford** | 0.5116 | 0.7 | 21 | Jackson | -0.245 | 0.4 | 72 |
| Knox | 0.503 | 0.69 | 22 | DeKalb | -0.247 | 0.4 | 73 |
| Pope | 0.4883 | 0.68 | 23 | Jersey | -0.265 | 0.4 | 74 |
| Tazewell | 0.4795 | 0.67 | 24 | Bureau | -0.312 | 0.39 | 75 |
| Fulton | 0.4785 | 0.67 | 25 | Kankakee** | -0.328 | 0.39 | 76 |
| Massac* | 0.4569 | 0.66 | 26 | McDonough | -0.351 | 0.38 | 77 |
| Williamson | 0.4227 | 0.66 | 27 | Macon* | -0.352 | 0.38 | 78 |
| Montgomery*, ** | 0.4042 | 0.65 | 28 | Warren | -0.4 | 0.37 | 79 |
| Cumberland | 0.3339 | 0.65 | 29 | Clay* | -0.419 | 0.37 | 80 |
| LaSalle | 0.333 | 0.64 | 30 | Logan*, ** | -0.427 | 0.36 | 81 |
| White** | 0.3279 | 0.64 | 31 | Menard | -0.462 | 0.35 | 82 |
| Calhoun** | 0.3242 | 0.64 | 32 | Rock Island | -0.468 | 0.34 | 83 |
| Johnson*, ** | 0.2938 | 0.64 | 33 | JoDaviess | -0.476 | 0.34 | 84 |
| Union** | 0.2795 | 0.64 | 34 | Sangamon | -0.586 | 0.34 | 85 |
| Wayne | 0.2717 | 0.63 | 35 | Piatt | -0.648 | 0.31 | 86 |
| Clark | 0.2351 | 0.63 | 36 | St Clair | -0.685 | 0.3 | 87 |
| Moultrie | 0.2332 | 0.63 | 37 | McLean | -0.686 | 0.29 | 88 |
| Jefferson | 0.2116 | 0.62 | 38 | Peoria | -0.698 | 0.26 | 89 |
| Edgar | 0.1789 | 0.61 | 39 | Winnebago | -0.699 | 0.24 | 90 |
| Shelby | 0.1307 | 0.61 | 40 | Woodford | -0.723 | 0.24 | 91 |
| Madison | 0.129 | 0.61 | 41 | Boone | -0.797 | 0.22 | 92 |
| Livingston | 0.1264 | 0.61 | 42 | Grundy | -0.843 | 0.21 | 93 |
| Marshall | 0.1186 | 0.61 | 43 | DeWitt*, ** | -0.92 | 0.2 | 94 |
| Schuyler*, ** | 0.1165 | 0.6 | 44 | Monroe | -1.029 | 0.07 | 95 |
| Ford | 0.1109 | 0.59 | 45 | McHenry | -1.127 | 0.06 | 96 |
| Scott | 0.1095 | 0.59 | 46 | Coles | -1.374 | 0.04 | 97 |
| Clinton | 0.1005 | 0.59 | 47 | Kendall | -1.417 | 0.04 | 98 |
| Richland | 0.0832 | 0.58 | 48 | Lake | -1.434 | 0.04 | 99 |
| Lee | 0.0745 | 0.57 | 49 | Champaign | -1.459 | 0.03 | 100 |
| Henderson | 0.0619 | 0.56 | 50 | Kane | -1.834 | 0.03 | 101 |
| Henry | 0.0503 | 0.56 | 51 | DuPage | -2.208 | 0.03 | 102 |

^*^ Top 20 counties have the highest age-adjusted lung cancer incidence rates (2014-2018)

^**^ Top 20 counties have the highest age-adjusted lung cancer death rates (2014-2018)

**More Details regarding AHP Procedure**

**Step 1: Recognizing then risk factors**

We performed a systematic review to determine the main modifiable lung cancer risk factors. Also, we performed a meta-analysis to evaluate the quantitate association of each of the risk factors in the causation of lung cancer (See Methods)

**Step 2: Create the comparison matrix and calculate then weights**

1. To make comparisons, we'll need a numerical scale that shows how much more important or dominating one element is over another in terms of the criterion or attribute against which they're compared.
2. The scale is shown in Table 3. The scale is used to compare the relative consumption of main lung cancer modifiable risk factors. One compares smoking on the left with one on the right and answers the question: How many times, or how strong, is smoking in the causation of lung cancer than the one on the right? The relevant number from the scale for the judgment is then entered (See Table 4).
3. To make a collection of pairwise comparison matrices, each element at a higher level is used to compare the elements in the levels below it with the ones in the level immediately below it.
4. To weigh the priorities in the level, use the priorities derived from the comparisons. Carry out the same procedure for each piece. Then add the weighed values for each element on the level below to get the overall or global priority.
5. Continue considering and adding until the final priorities of the options at the bottom most level is established. The priorities (obtained in exact form by raising the matrix to large powers and summing each row and dividing by the total sum of all the rows, or roughly by adding each row of the matrix and dividing by their total) can be calculated using Excel or MATLAB.

**Table 3** The scale and deﬁnition of the assessment matrix.

| Distinguish  Scale | 1 | 3 | 5 | 7 | 9 | 2,4,6,8 |
| --- | --- | --- | --- | --- | --- | --- |
| Deﬁnition | Equal importance | Weak importance | Obvious importance | Intensely importance | Extreme importance | Intermediate  value |
| Explanation | Two activities contribute equally to the objective | Experience  and judgement slightly favour one activity over another | Experience and judgement strongly favour  one activity over another | An activity is favoured very strongly over  another; its dominance demonstrated in practice | The evidence favouring one activity over another  is of the highest possible order of affirmation |  |

**Table 4** The pairwise comparison matrix

|  | Smoking | Indoor air pollution | Occupational exposure | Second hand smoking | Alcohol consumption | Radon | Outdoor air pollution |
| --- | --- | --- | --- | --- | --- | --- | --- |
| Smoking | 1.00 | 5.00 | 6.00 | 6.00 | 7.00 | 7.00 | 8.00 |
| Indoor air pollution | 0.20 | 1.00 | 3.00 | 3.00 | 4.00 | 5.00 | 6.00 |
| Occupational exposure | 0.20 | 0.33 | 1.00 | 3.00 | 4.00 | 4.00 | 5.00 |
| Second hand smoking | 0.17 | 0.33 | 0.33 | 1.00 | 2.00 | 3.00 | 4.00 |
| Alcohol consumption | 0.14 | 0.25 | 0.25 | 0.50 | 1.00 | 3.00 | 4.00 |
| Radon | 0.14 | 0.20 | 0.25 | 0.33 | 0.33 | 1.00 | 3.00 |
| Outdoor air pollution | 0.13 | 0.17 | 0.20 | 0.25 | 0.25 | 0.33 | 1.00 |

**References**

1. Remen, T., et al., *Risk of lung cancer in relation to various metrics of smoking history: a case-control study in Montreal.* BMC cancer, 2018. **18**(1): p. 1-12.

2. Stellman, S.D., et al., *Smoking and lung cancer risk in American and Japanese men: an international case-control study.* Cancer Epidemiology and Prevention Biomarkers, 2001. **10**(11): p. 1193-1199.

3. Risch, H.A., et al., *Are female smokers at higher risk for lung cancer than male smokers? A case-control analysis by histologic type.* American journal of epidemiology, 1993. **138**(5): p. 281-293.

4. Katsouyanni, K., et al., *A case-control study of air pollution and tobacco smoking in lung cancer among women in Athens.* Preventive Medicine, 1991. **20**(2): p. 271-278.

5. Wakai, K., et al., *Tobacco smoking and lung cancer risk: an evaluation based on a systematic review of epidemiological evidence among the Japanese population.* Japanese journal of clinical oncology, 2006. **36**(5): p. 309-324.

6. Boffetta, P., et al., *Cigar and pipe smoking and lung cancer risk: a multicenter study from Europe.* Journal of the National Cancer Institute, 1999. **91**(8): p. 697-701.

7. Armadans-Gil, L., et al., *Cigarette smoking and male lung cancer risk with special regard to type of tobacco.* International journal of epidemiology, 1999. **28**(4): p. 614-619.

8. Luqman, M., et al., *Risk factors for lung cancer in the Pakistani population.* Asian Pacific Journal of Cancer Prevention, 2014. **15**(7): p. 3035-3039.

9. Brownson, R.C., J.C. Chang, and J.R. Davis, *Gender and histologic type variations in smoking-related risk of lung cancer.* Epidemiology, 1992: p. 61-64.

10. Agudo, A., et al., *Lung cancer and cigarette smoking in women: A multicenter case‐control study in Europe.* International journal of cancer, 2000. **88**(5): p. 820-827.

11. Behera, D. and T. Balamugesh, *Indoor air pollution as a risk factor for lung cancer in women.* JAPI, 2005. **53**: p. 190-192.

12. Krewski, D., et al., *A combined analysis of North American case-control studies of residential radon and lung cancer.* Journal of Toxicology and Environmental Health, Part A, 2006. **69**(7-8): p. 533-597.

13. H. Lubin, J., *Studies of radon and lung cancer in North America and China.* Radiation protection dosimetry, 2003. **104**(4): p. 315-319.

14. Lubin, J.H., et al., *Risk of lung cancer and residential radon in China: pooled results of two studies.* International Journal of Cancer, 2004. **109**(1): p. 132-137.

15. Lorenzo-González, M., et al., *Lung cancer and residential radon in never-smokers: A pooling study in the Northwest of Spain.* Environmental research, 2019. **172**: p. 713-718.

16. Kreienbrock, L., et al., *Case-control study on lung cancer and residential radon in western Germany.* American Journal of Epidemiology, 2001. **153**(1): p. 42-52.

17. Lagarde, F., et al., *Residential radon and lung cancer among never-smokers in Sweden.* Epidemiology, 2001: p. 396-404.

18. Brenner, D.R., et al., *Lung cancer risk in never-smokers: a population-based case-control study of epidemiologic risk factors.* BMC cancer, 2010. **10**(1): p. 1-9.

19. Cassidy, A., et al., *Occupational exposure to crystalline silica and risk of lung cancer: a multicenter case-control study in Europe.* Epidemiology, 2007: p. 36-43.

20. Brüske-Hohlfeld, I., et al., *Occupational lung cancer risk for men in Germany: results from a pooled case-control study.* American journal of epidemiology, 2000. **151**(4): p. 384-395.

21. Pintos, J., et al., *Occupational exposure to asbestos and man-made vitreous fibers, and risk of lung cancer: evidence from two case-control studies in Montreal, Canada.* Journal of occupational and environmental medicine, 2008. **50**(11): p. 1273-1281.

22. Villeneuve, P.J., et al., *Occupational exposure to asbestos and lung cancer in men: evidence from a population-based case-control study in eight Canadian provinces.* BMC cancer, 2012. **12**(1): p. 1-10.

23. Stayner, L., et al., *Lung cancer risk and workplace exposure to environmental tobacco smoke.* American Journal of Public Health, 2007. **97**(3): p. 545-551.

24. Hackshaw, A.K., M.R. Law, and N.J. Wald, *The accumulated evidence on lung cancer and environmental tobacco smoke.* Bmj, 1997. **315**(7114): p. 980-988.

25. Seki, T., et al., *Cigarette smoking and lung cancer risk according to histologic type in Japanese men and women.* Cancer science, 2013. **104**(11): p. 1515-1522.

26. Kabat, G.C., S.D. Stellman, and E.L. Wynder, *Relation between exposure to environmental tobacco smoke and lung cancer in lifetime nonsmokers.* American journal of epidemiology, 1995. **142**(2): p. 141-148.

27. Nyberg, F., et al., *Environmental tobacco smoke and lung cancer in nonsmokers: does time since exposure play a role?* Epidemiology, 1998: p. 301-308.

28. Sobue, T., *Association of indoor air pollution and lifestyle with lung cancer in Osaka, Japan.* International Journal of Epidemiology, 1990. **19**(Supplement_1): p. S62-S66.

29. Jee, S.H., H. Ohrr, and I.S. Kim, *Effects of husbands' smoking on the incidence of lung cancer in Korean women.* International Journal of Epidemiology, 1999. **28**(5): p. 824-828.

30. Brownson, R.C., et al., *Passive smoking and lung cancer in nonsmoking women.* American Journal of Public Health, 1992. **82**(11): p. 1525-1530.

31. Ko, Y.-C., et al., *Risk factors for primary lung cancer among non-smoking women in Taiwan.* International journal of epidemiology, 1997. **26**(1): p. 24-31.

32. Ribeiro, A.G., et al., *Incidence and mortality risk for respiratory tract cancer in the city of São Paulo, Brazil: Bayesian analysis of the association with traffic density.* Cancer epidemiology, 2018. **56**: p. 53-59.

33. Vineis, P., et al., *Air pollution and risk of lung cancer in a prospective study in Europe.* International journal of cancer, 2006. **119**(1): p. 169-174.

34. Hystad, P., et al., *Long-term residential exposure to air pollution and lung cancer risk.* Epidemiology, 2013: p. 762-772.

35. Vineis, P., et al., *Lung cancers attributable to environmental tobacco smoke and air pollution in non-smokers in different European countries: a prospective study.* Environmental Health, 2007. **6**(1): p. 1-7.

36. Pope Iii, C.A., et al., *Lung cancer, cardiopulmonary mortality, and long-term exposure to fine particulate air pollution.* Jama, 2002. **287**(9): p. 1132-1141.

37. Nafstad, P., et al., *Lung cancer and air pollution: a 27 year follow up of 16 209 Norwegian men.* Thorax, 2003. **58**(12): p. 1071-1076.

38. Abbey, D.E., et al., *Long-term inhalable particles and other air pollutants related to mortality in nonsmokers.* American journal of respiratory and critical care medicine, 1999. **159**(2): p. 373-382.

39. Nyberg, F., et al., *Urban air pollution and lung cancer in Stockholm.* Epidemiology, 2000: p. 487-495.

40. Raspanti, G.A., et al., *Household air pollution and lung cancer risk among never-smokers in Nepal.* Environmental research, 2016. **147**: p. 141-145.

41. Vermeulen, R., et al., *Constituents of household air pollution and risk of lung cancer among never-smoking women in Xuanwei and Fuyuan, China.* Environmental health perspectives, 2019. **127**(9): p. 097001.

42. Seow, A., et al., *Fumes from meat cooking and lung cancer risk in Chinese women.* Cancer Epidemiology and Prevention Biomarkers, 2000. **9**(11): p. 1215-1221.

43. Bruce, N., et al., *Does household use of biomass fuel cause lung cancer? A systematic review and evaluation of the evidence for the GBD 2010 study.* Thorax, 2015. **70**(5): p. 433-441.

44. Kleinerman, R., et al., *Lung cancer and indoor air pollution in rural China.* Annals of epidemiology, 2000. **10**(7): p. 469.

45. Ko, Y.-C., et al., *Chinese food cooking and lung cancer in women nonsmokers.* American journal of epidemiology, 2000. **151**(2): p. 140-147.

46. Bagnardi, V., et al., *Alcohol consumption and lung cancer risk in the Environment and Genetics in Lung Cancer Etiology (EAGLE) study.* American journal of epidemiology, 2010. **171**(1): p. 36-44.

47. Brenner, D.R., et al., *Alcohol consumption and lung cancer risk: A pooled analysis from the International Lung Cancer Consortium and the SYNERGY study.* Cancer epidemiology, 2019. **58**: p. 25-32.

48. Bandera, E.V., et al., *Alcohol consumption and lung cancer in white males.* Cancer Causes & Control, 1992. **3**(4): p. 361-369.

49. Ruano-Ravina, A., A. Figueiras, and J. Barros-Dios, *Type of wine and risk of lung cancer: a case-control study in Spain.* Thorax, 2004. **59**(11): p. 981-985.

50. Boffetta, P., et al., *Risk of cancers of the lung, head and neck in patients hospitalized for alcoholism in Sweden.* British journal of cancer, 2001. **85**(5): p. 678-682.

51. Carpenter, C.L., H. Morgenstern, and S.J. London, *Alcoholic beverage consumption and lung cancer risk among residents of Los Angeles County.* The Journal of nutrition, 1998. **128**(4): p. 694-700.

52. Hu, J., et al., *Risk factors for lung cancer among Canadian women who have never smoked.* Cancer detection and prevention, 2002. **26**(2): p. 129-138.

53. Benedetti, A., M.-E. Parent, and J. Siemiatycki, *Consumption of alcoholic beverages and risk of lung cancer: results from two case–control studies in Montreal, Canada.* Cancer Causes & Control, 2006. **17**(4): p. 469-480.
